# Supplementary material for: High-throughput mechanical phenotyping and transcriptomics of single cells
Source: Nat Commun. 2024 May 17;15:3812. doi: 10.1038/s41467-024-48088-5 (PMC11101642; doi:10.1038/s41467-024-48088-5)
Supplement: Supplementary file 1 — Supplementary Information [file 41467_2024_48088_MOESM1_ESM.pdf]

Supplementary information

High-throughput mechanical phenotyping and transcriptomics of single cells

Akifumi Shiomi[1], Taikopaul Kaneko[1], Kaori Nishikawa[1], Arata Tsuchida[1], Takashi Isoshima[1], Mayuko Sato[2], Kiminori Toyooka[2], Kentaro Doi[3], Hidekazu Nishikii[4], and Hirofumi Shintaku[1, 5]
[1]Cluster for Pioneering Research, RIKEN
[2]Center for Sustainable Resource Science, RIKEN
[3]Department of Mechanical Engineering, Toyohashi University of Technology
[4]Faculty of Medicine, University of Tsukuba
[5]Institute for Life and Medical Sciences, Kyoto University

Corresponding author's email: shintaku@infront.kyoto-u.ac.jp

Supplementary Figures 1-13

Supplementary Tables 1-3

Supplementary Methods

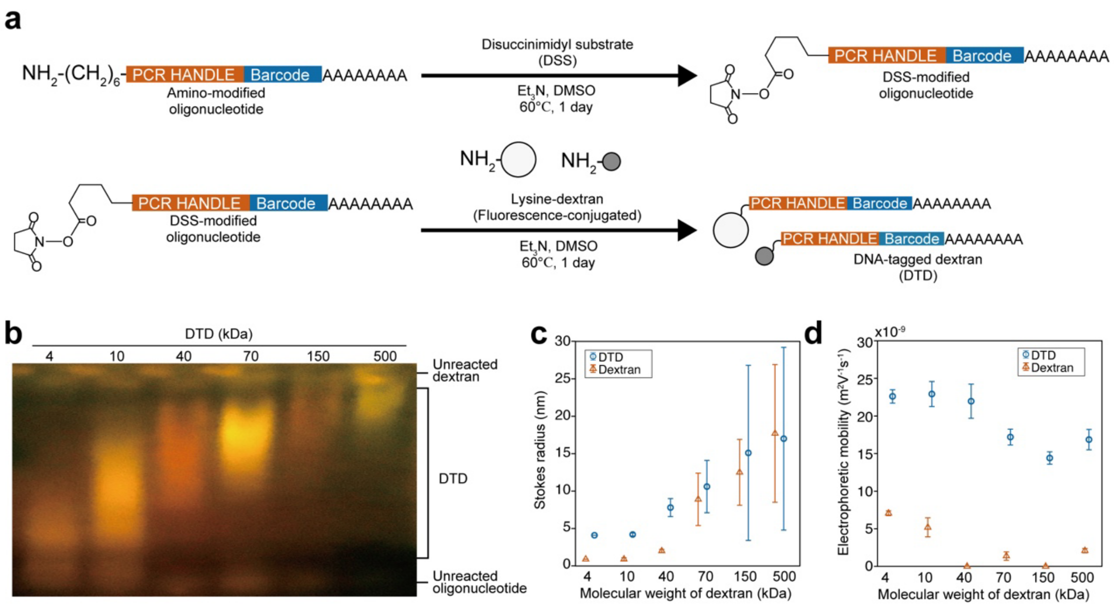

Supplementary Figure 1. **Synthesis of DNA-tagged dextran (DTD).** **a** Synthesis scheme of DTD. **b** Photograph of agarose gel electrophoresis to separate synthesized DTDs from unreacted oligonucleotide and dextran. DTD and unreacted oligonucleotide were visualized by SYBR Green II. **c** Stokes radii of dextran and DTD measured by 100 times dynamic light scattering. Results are represented as average ± SD. **d** The electrophoretic mobility of dextran and DTD measured by a microfluidic approach. Results are represented as average ± SE (Dextran: n = 3; 4, 10, 40, and 500 kDa DTD: n = 4; 70 and 150 kDa DTD: n = 5 independent experiences). Source data are provided as a Source Data file.

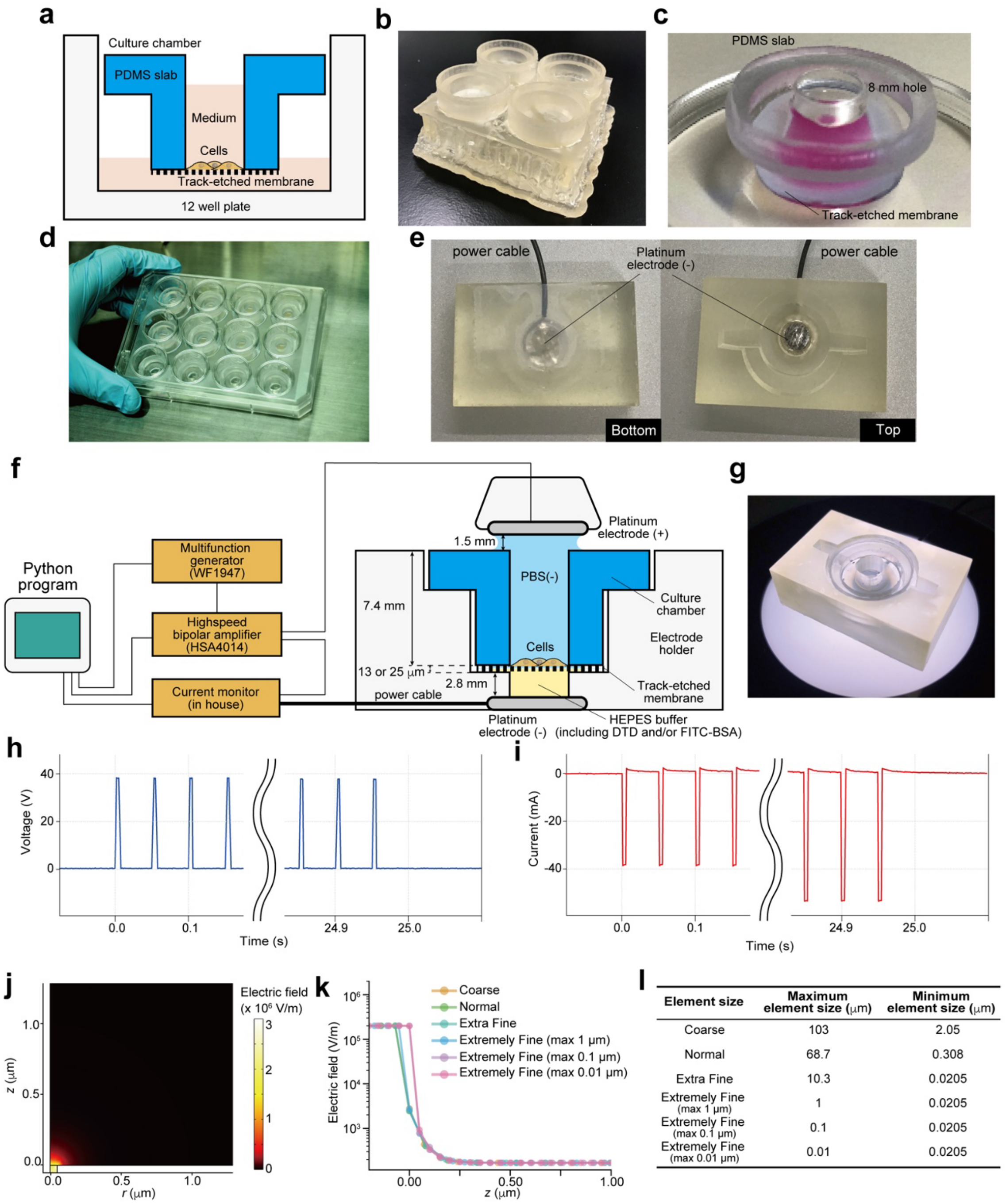

Element size	Maximum element size (µm)	Minimum element size (µm)
Coarse	103	2.05
Normal	68.7	0.308
Extra Fine	10.3	0.0205
Extremely Fine (max 1 µm)	1	0.0205
Extremely Fine (max 0.1 µm)	0.1	0.0205
Extremely Fine (max 0.01 µm)	0.01	0.0205

Supplementary Figure 2. **A system for ELASTomics protocol. a** Schematic image of a culture chamber. The cells seeded on a track etched membrane were cultured by immersing the culture chamber in a culture medium. **b** Photograph of mould for a culture chamber. **c** Photograph of a culture chamber filled with a culture medium. **d** Photograph of culture chambers in a 12-well plate. **e** Photographs of the bottom and top side of an electrode holder. **f** Schematic diagram of the nanopore-electroporation device. **g** Photograph of a culture chamber installed in the electrode holder. **h, i** Applied voltage and current experimentally measured during the nanopore-electroporation. **j** The electric field focused around the nanopore. The electric field was computed in cylindrical coordination using COMSOL Multiphysics software. The colour indicates the magnitude of the electric field. **k, l** Numerically computed electric field along the z-axis with various mesh sizes. (*j-l*) The track-etched membrane is modelled as an axisymmetric cylinder with 25-µm height and 100 nm in diameter.

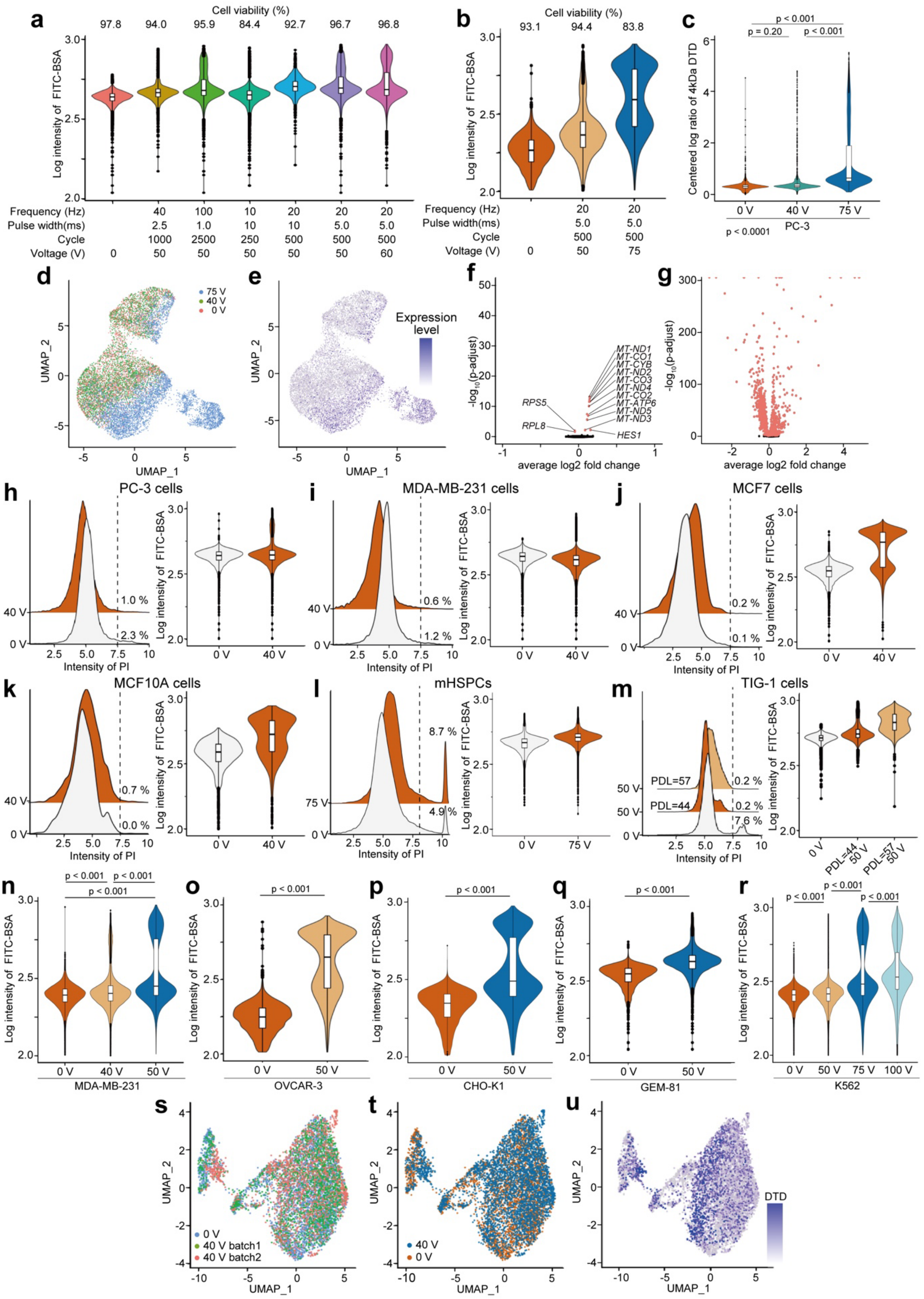
a
Cell viability (%)
97.8 94.0 95.9 84.4 92.7 96.7 96.8
Log intensity of FITC-BSA
Frequency (Hz) 40 100 10 20 20 20
Pulse width(ms) 2.5 1.0 10 10 5.0 5.0
Cycle 1000 2500 250 500 500 500
Voltage (V) 0 50 50 50 50 50 60
b
Cell viability (%)
93.1 94.4 83.8
Log intensity of FITC-BSA
Frequency (Hz) 20 20
Pulse width(ms) 5.0 5.0
Cycle 500 500
Voltage (V) 0 50 75
c
Centered log ratio of 4kDa DTD
p < 0.001
p = 0.20
p < 0.001
0 V 40 V 75 V
p < 0.0001
PC-3
d
75 V
40 V
0 V
UMAP_2
UMAP_1
e
Expression level
UMAP_2
UMAP_1
f
-log10(p-adjust)
MT-ND1
MT-CO1
MT-CYB
MT-ND2
MT-CO3
MT-ND4
MT-CO2
MT-ATP6
MT-ND5
MT-ND3
RPS5
RPL8
HES1
average log2 fold change
g
-log10(p-adjust)
average log2 fold change
h
PC-3 cells
40 V
0 V
1.0 %
2.3 %
Intensity of PI
Log intensity of FITC-BSA
0 V 40 V
i
MDA-MB-231 cells
40 V
0 V
0.6 %
1.2 %
Intensity of PI
Log intensity of FITC-BSA
0 V 40 V
j
MCF7 cells
40 V
0 V
0.2 %
0.1 %
Intensity of PI
Log intensity of FITC-BSA
0 V 40 V
k
MCF10A cells
40 V
0 V
0.7 %
0.0 %
Intensity of PI
Log intensity of FITC-BSA
0 V 40 V
l
mHSPCs
75 V
0 V
8.7 %
4.9 %
Intensity of PI
Log intensity of FITC-BSA
0 V 75 V
m
TIG-1 cells
50 V PDL=57
50 V PDL=44
0 V
0.2 %
0.2 %
7.6 %
Intensity of PI
Log intensity of FITC-BSA
0 V
PDL=44 50 V
PDL=57 50 V
n
p < 0.001
p < 0.001
p < 0.001
Log intensity of FITC-BSA
0 V 40 V 50 V
MDA-MB-231
o
p < 0.001
Log intensity of FITC-BSA
0 V 50 V
OVCAR-3
p
p < 0.001
Log intensity of FITC-BSA
0 V 50 V
CHO-K1
q
p < 0.001
Log intensity of FITC-BSA
0 V 50 V
GEM-81
r
p < 0.001
p < 0.001
p < 0.001
Log intensity of FITC-BSA
0 V 50 V 75 V 100 V
K562
s
0 V
40 V batch1
40 V batch2
UMAP_2
UMAP_1
t
40 V
0 V
UMAP_2
UMAP_1
u
DTD
UMAP_2
UMAP_1

Supplementary Figure 3. **Optimization of nanopore electroporation for ELASTomics. a** Intensity of FITC-BSA imported into HeLa cells by nanopore-electroporation with different electric pulses (n = 2014 to 8196 independent cells). The percentage indicates the cell viability. **b** Intensity of FITC-BSA imported into HeLa cells by nanopore-electroporation with different voltages (0V, n = 3000; 50 V, n = 7028; 75 V, n =3856). The percentage indicates the cell viability. Similar results were observed in independent duplicate experiments. **c** Centred log ratios of 4 kDa DTD imported into PC-3 cells with different applied voltages. **d, e** Uniform manifold approximation and projection (UMAP) of PC-3 cells. The colour indicates the applied voltages (blue: 75 V; green: 40 V; red: 0 V) (*d*) and centred log ratios of 4 kDa DTD imported into cells by nanopore electroporation (*e*). **f, g** Volcano plots of differentially expressed genes between 0V and 40V (*f*) or 75 V (*g*). Red points are the genes with adjusted p-values < 0.05. **h-m** Intensity of propidium iodide (PI) (*left*) and FITC-BSA imported by nanopore-electroporation (*right*) with the optimised electric pulses for ELASTomics in PC-3 (n = 21,575 to 25,460 independent cells), MDA-MB-231 (n = 16,640 to 31,573 independent cells), MCF7 (n = 11,164 to 12,919 independent cells), and MCF10A cells (n = 1925 to 2944 independent cells) (40 V), mouse haematopoietic stem/progenitor cells (mHSPCs) (n = 41,784 to 73,190 independent cells) (75 V), and TIG-1 cells at population doubling levels (PDL = 44 or 57) (n = 9126 to 10,023 independent cells) (50V). The percentage in the left figure indicates the fraction of dead cells. Similar results were observed in independent duplicate experiments. **n-r** Fluorescence intensity of imported FITC-BSA by nanopore electroporation in MDA-MB-231 (*n*; adherent cells, human), OVCAR-3 (*o*; adherent cells, human), CHO-K1 (*p*; adherent cells, Chinese hamster), GEM-81 cells (*q*; adherent cells, goldfish), and K562 (*r*; suspension cells, human) (n = 8066 to 20,828 independent cells). Similar results were observed in independent duplicate experiments. **s-u** Uniform manifold approximation and projection (UMAP) of MCF10A cells after integration. The colour indicates the experimental batches (blue: 0 V; green: 40V batch 1; red: 40 V batch2) (*s*), the applied voltages (red: 0 V; blue: 40 V) (*t*), and centred log ratios of 4 kDa DTD imported into cells by nanopore-electroporation (*u*). For boxplots overlaid on violin plots, the centre lines are the median, the box indicates the first and third quartiles, whiskers are minimum/maximum values excluding outliers, and dots are outliers. The P values (*p*) are indicated in the graph (o-q: two-tailed Student's t-test; c, n, and r: Tukey's t-test).

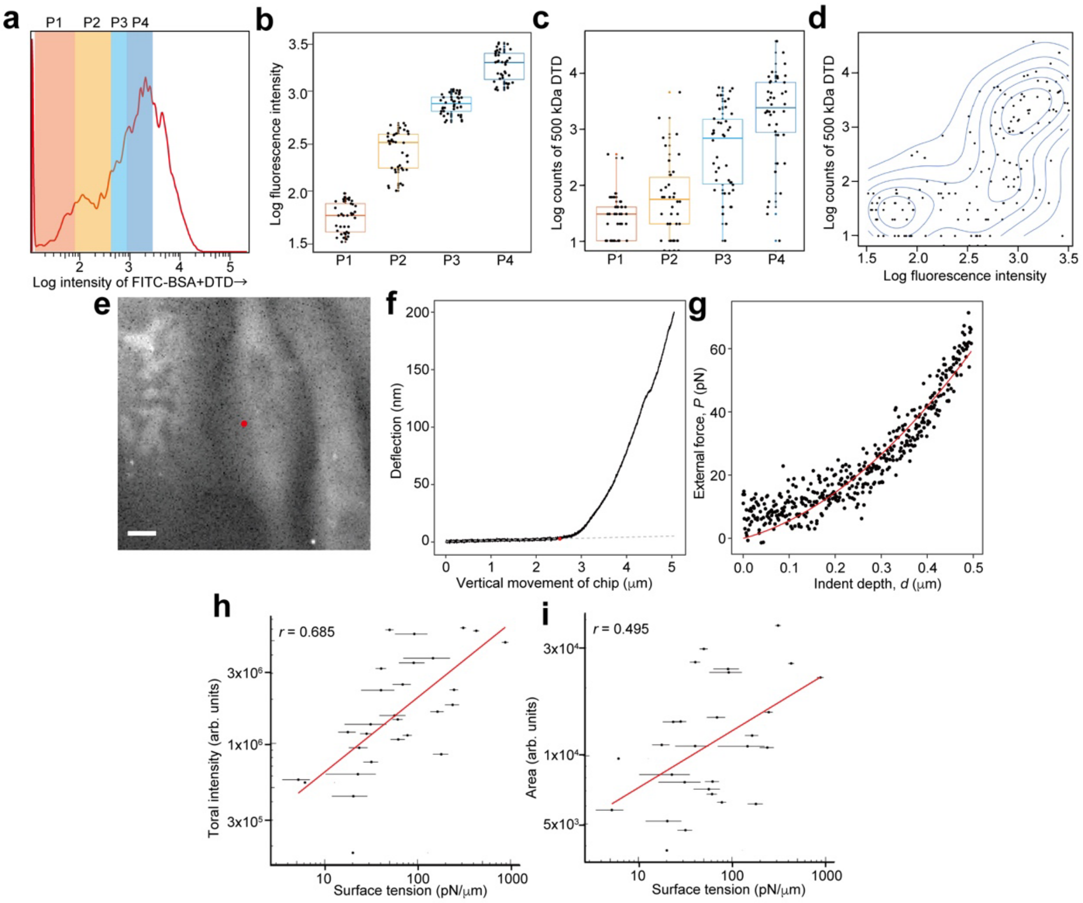

Supplementary Figure 4. **Correlation between FITC-BSA, DTD counts, and cell surface tension. a** Integrated fluorescence intensity of DTD and FITC-BSA imported into MCF10A cells measured by flow cytometry. Coloured boxes (P1-P4) are the gates for sorting cells (P1: n = 37; P2: n = 38; P3: n = 42; P4: n = 40 independent cells). **b** Fluorescence intensities of sorted cells summarized by the P1-P4 gates. **c** The log counts of 500 kDa DTD quantified by RNA-sequencing. **d** Correlation between fluorescence intensity (horizontal axis) measured by flow cytometry and the log counts of 500 kDa DTD by RNA-seq (vertical axis). Contour lines are the iso-density lines. **e** Fluorescence image of a MCF10A cell labeled with FITC-BSA by nanopore-electroporation. The red dot indicates a measurement point (*f* and *g*) by atomic force microscopy (AFM). Scale bar, 10 μm. **f** An example of a force curve measured by AFM. The broken line indicates the horizontal line to define the contact point (red dot). **g** Zoom-up view of the force curve around the contact point. The red line indicates the fitting curve to the data to estimate cell surface tension. **h, i** Comparison of total intensities of FITC-BSA (*h*) or cell adhesion areas (*i*) versus the cell surface tension measured by atomic force microscopy in each nanopore-electroporated MCF10A cell (n = 28 independent cells examined in four independent experiments). The coefficients of Pearson's correlation (two-tailed) were respectively $r = 0.685$ (*h*) and $r = 0.495$ (*i*). Error bars of surface tension were presented as mean ± SE (n = 2 to 44 per cell). The red lines represent the results of regression. For boxplot (b and c), the centre line is the median, the box indicates the first and third quartiles, whiskers are minimum/maximum values excluding outliers, and dots are individual data. Source data are provided as a Source Data file.

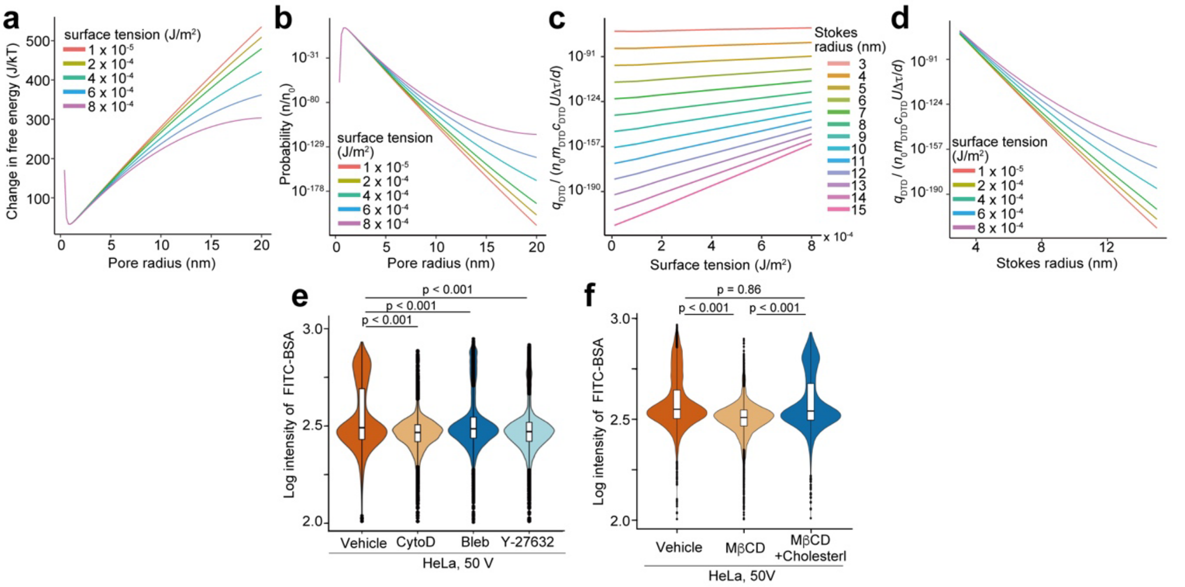

Supplementary Figure 5. **Theoretical and experimental analysis of the effect of cell surface tension on nanopores formed by nanopore electroporation. a-d** Theoretical predictions of change in free energy for the pore formation, the probability density distribution of the radius of a pore, and the relative permeability of molecules with various Stokes radii. (*a*) Change in free energy for pore formation as a function of the radius of a pore in a plasma membrane. (*b*) The probability density distribution of a pore formed in a plasma membrane at various cell surface tensions. (*c*) The relative permeability of imported molecules at various cell surface tension. (*d*) The relative permeability of imported molecules as a function of Stokes radii. **e, f** Intensity of FITC-BSA imported into HeLa cells by nanopore electroporation with 50 V. (*e*) HeLa cells treated with vehicle, cytochalasin D (CytoD), blebbistatin (Bleb), or Y-27632 (vehicle: n = 6012; CytoD: n =9828; Bleb: n = 9874; Y-27632: n = 8812 independent cells). (*f*) HeLa cells treated with vehicle and methyl-β-cyclodextrin (MβCD) with or without cholesterol. (vehicle: n = 8208; MβCD: n = 8239; MβCD+Cholesterol: n = 8276 independent cells). The P values (*p*) are indicated in the graph (Tukey's t-test). Similar results were observed in independent duplicate experiments. For boxplots overlaid on violin plots, the centre lines are the median, the box indicates the first and third quartiles, whiskers are minimum/maximum values excluding outliers, and dots are outliers.

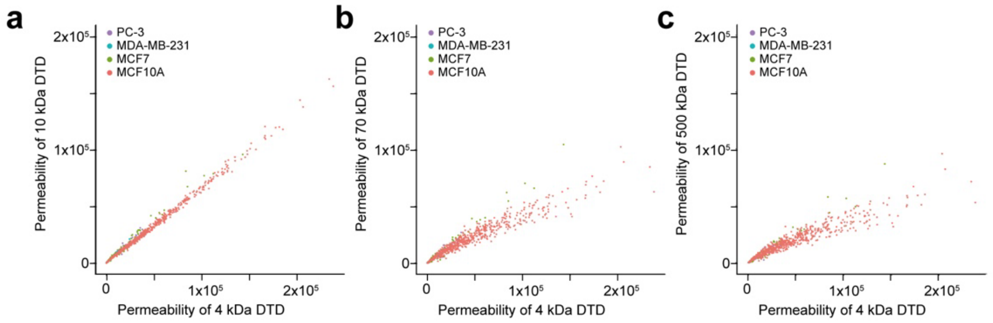

Supplementary Figure 6. **Effect of the molecular weight on normalized DTD counts. a-c** Permeability in four cell lines (purple: PC-3; blue: MDA-MB-231; green: MCF7; red: MCF10A). The permeability of DTDs is defined as normalized counts by concentration and the mobility of each DTD.

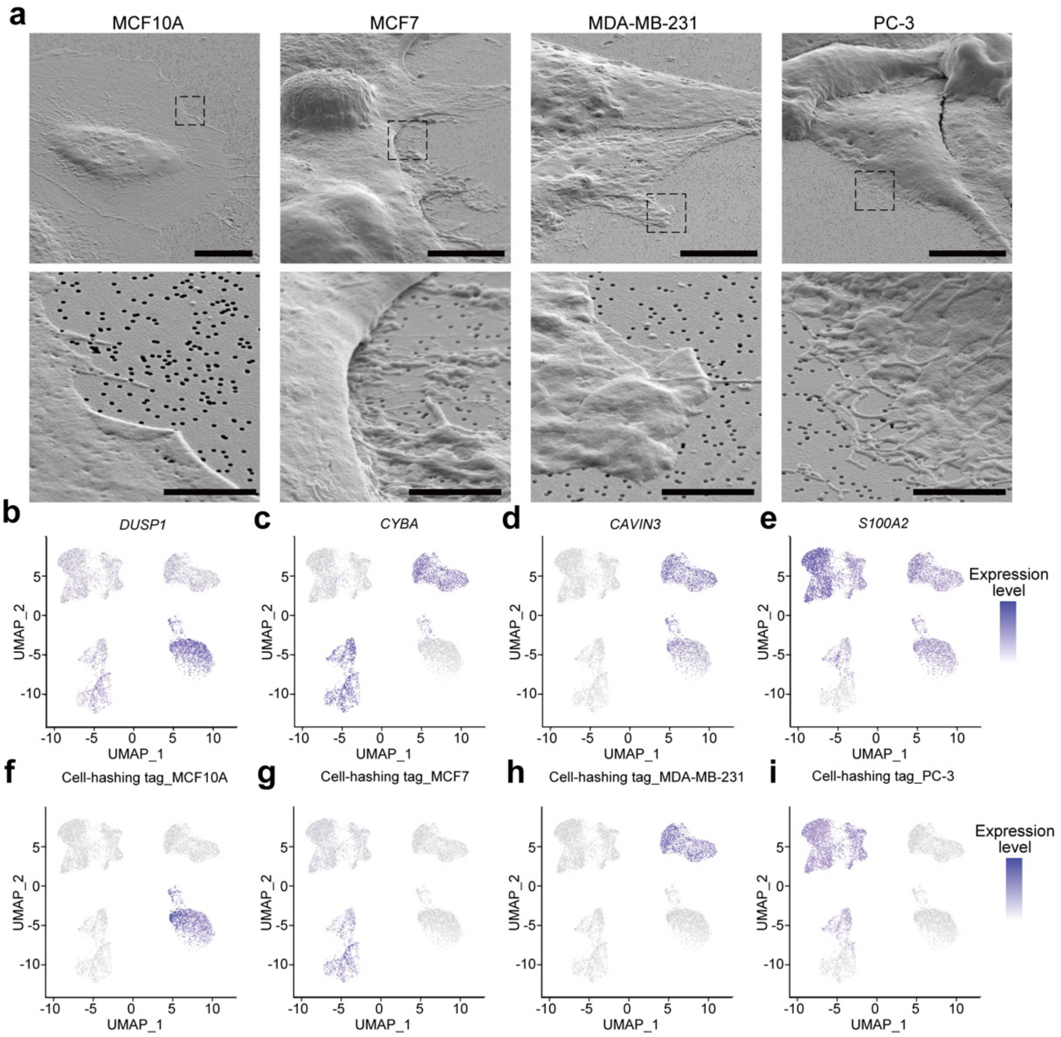

Supplementary Figure 7. **ELASTomics analysis of cancer cell lines. a** Scanning electron microscope images of MCF10A, MCF7, MDA-MB-231, and PC-3 cells cultured on track-etched membranes. Lower panels are zoom-up views of respective boxes in the upper panels. Scale bars, 10 µm (upper) or 2 µm (lower). **b-i** Uniform manifold approximation and projection (UMAP) plot of four cell lines. The colour indicates the expression of marker genes (*DUSP1*, *CYBA*, *CAVIN3*, *S100A2*) and the centred log ratio of cell-hashing tags. The hashing tag for PC-3 was susceptive to the background noise in i because of the low counts due to the low surface tension of PC-3 cells. However, the low specificity of the cell hashing tag did not detract from the identification of cell type and batch of clusters, because we identified the cell types based on the gene expression. (*a*) Similar results were observed in independent duplicate experiments imaged at different angles.

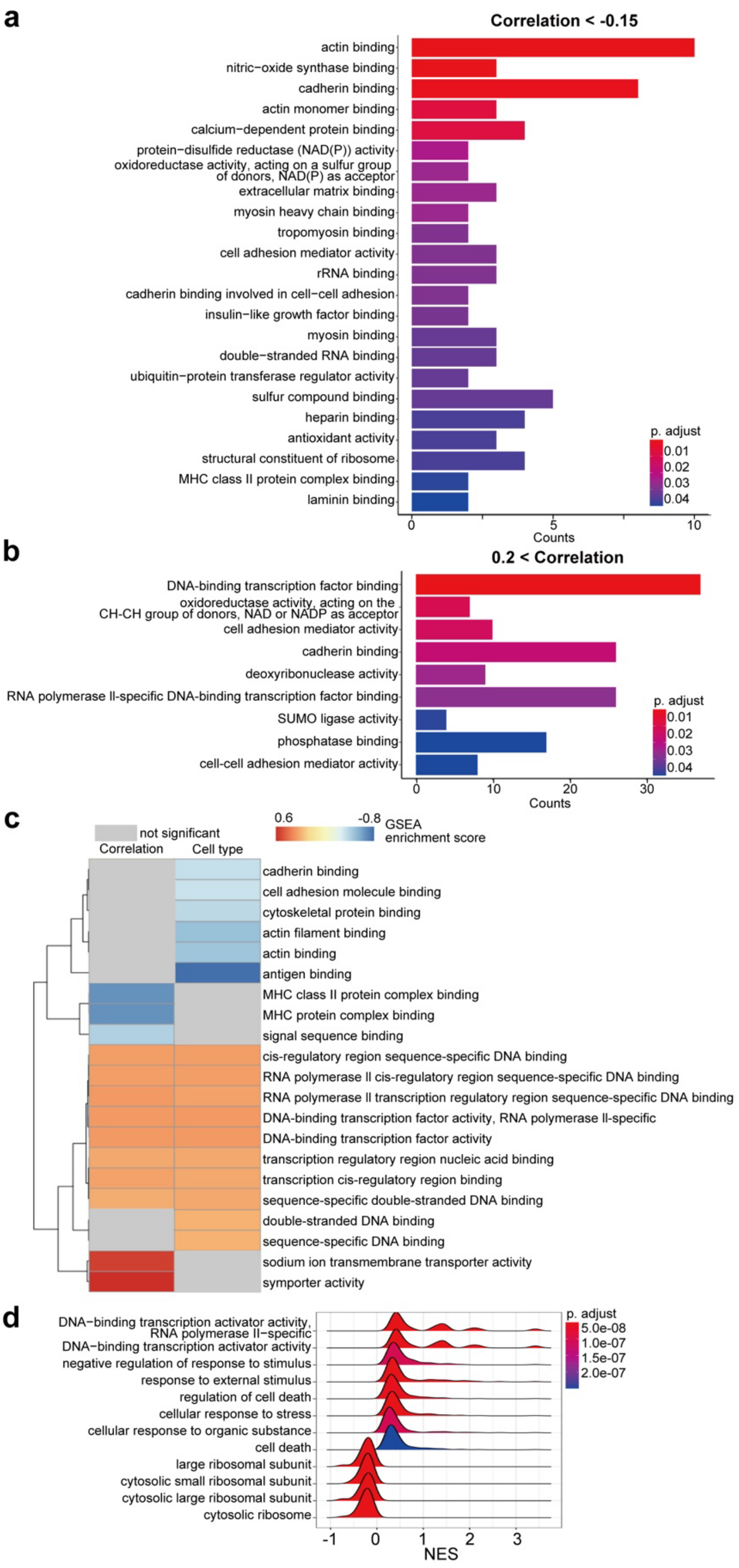

Supplementary Figure 8. **Gene ontology and gene set enrichment analysis on cancer cell lines. a, b** Gene ontology (GO) analysis on the gene sets negatively (*a*; correlation < -0.15) or positively (*b*; correlation > 0.2) correlated with the centred log ratios of 4 kDa DTD imported into MCF7 and MDA-MB-231 cells by nanopore-electroporation as shown in Fig. 1j. **c** Comparison of gene set enrichment analysis (GSEA) performed with the coefficient of correlation between the gene expression and centred log ratio of 4 kDa DTD against that with the fold change of the gene expression between MCF7 and MDA-MB-231 cells (grey: not significant). **d** GSEA performed with the change in the gene expression between MCF10A cells applied at 0 V and 40 V. NES, normalized enrichment score.

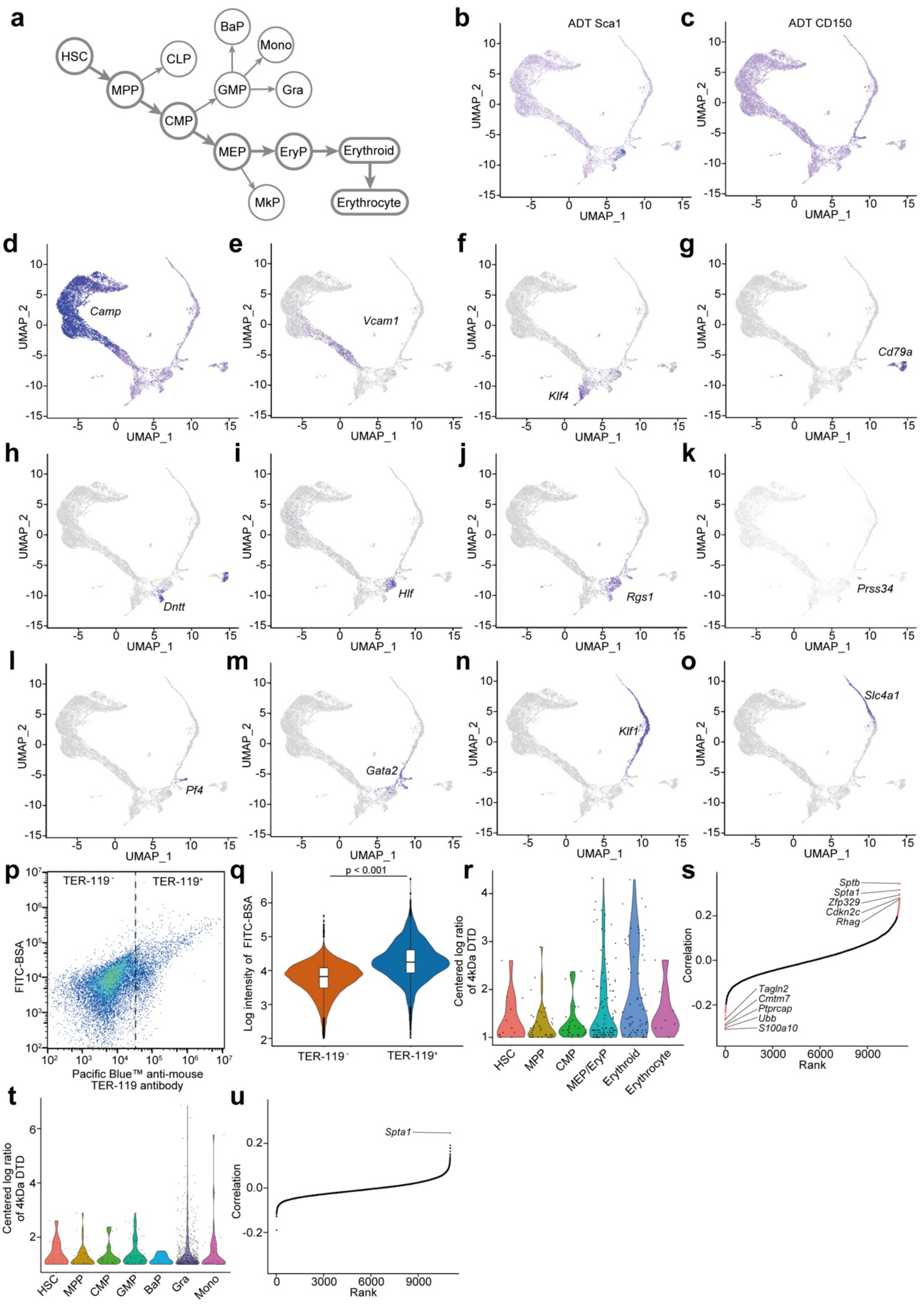

Supplementary Figure 9. **ELASTomics analysis of mouse hematopoietic stem/ progenitor cells (mHSPCs). a** Lineage tree of mHSPCs. The thick line indicates the differentiation pathway of the erythroid lineage. **b, c** Uniform manifold approximation and projection (UMAP) plot of mHSPCs. The colour indicates the antibody-derived tag (ADT) counts targeting Sca1 (b) and Cd150 (c). **d-o** UMAP plot of mHSPCs. The colour gradient indicates the expression level of marker genes (*Camp*: granulocyte (Gra); *Vcam1*: granulocyte-macrophage progenitor (GMP); *Klf4*: monocyte (Mono); *Cd79a*: B-cells; *Dntt*: common

lymphoid progenitor (CLP); *Hlf*: hematopoietic stem cell (HSC); *Rgs1*: multipotential progenitors (MPP); *Prss34:* basophil progenitors (BaP); *Pf4*: megakaryocyte progenitors (MkP); *Gata2*: common myeloid progenitor (CMP); *Klf1*: megakaryocyte-erythrocyte progenitors (MEP) and erythroid progenitors (EryP); *Slc4a1*: erythroid). **p** Flow cytometry analysis of c-kit+ bone marrow (BM) cells labelled with FITC-BSA by nanopore electroporation and PacificBlue™-conjugated antibody targeting TER-119, the surface marker of erythroid lineage. TER-119 positive (TER-119^{+}) or negative (TER-119^{-}) cells were defined as bordering on the intensity of Pacific Blue™ = 3 ×10^4. **q** Intensity of FITC-BSA imported by nanopore-electroporation in TER-119^{+} (n = 2457 independent cells) or TER-119^{-} (n = 14168 independent cells) cells. Similar results were observed in independent duplicate experiments. **r** The centred log ratio of 4 kDa DTD imported into cells along the erythroid lineage (HSC-MPP-CMP-MEP/EryP-Erythroid-Erythrocyte). Cells with low DTD counts (centred log ratio <1) were excluded in the visualization. **s** Quantile plot of genes sorted in the order of the coefficients of correlation between the centred log ratio of 4 kDa DTD and gene expression in erythroid lineage cells shown in (*r*). Red points indicate genes with Pearson's $|r| > 0.2$. **t** The centred log ratio of 4 kDa DTD in the myeloid lineage cells (HSC-MPP-CMP-GMP-BaP-Gra-Mono). Cells with low DTD counts (centred log ratio <1) were excluded in the visualization. **u** Quantile plot of genes sorted in the order of the coefficients of correlation between the centred log ratio of imported 4 kDa DTD and gene expression in the myeloid lineage cells shown in (*t*). Red dots indicate genes with Pearson's $|r| > 0.2$. (*q*) The P values (*p*) are indicated in the graph (two-tailed Student's t-test). For boxplots overlaid on violin plots, the centre lines are the median, the box indicates the first and third quartiles, whiskers are minimum/maximum values excluding outliers, and dots are outliers. Source data are provided as a Source Data file.

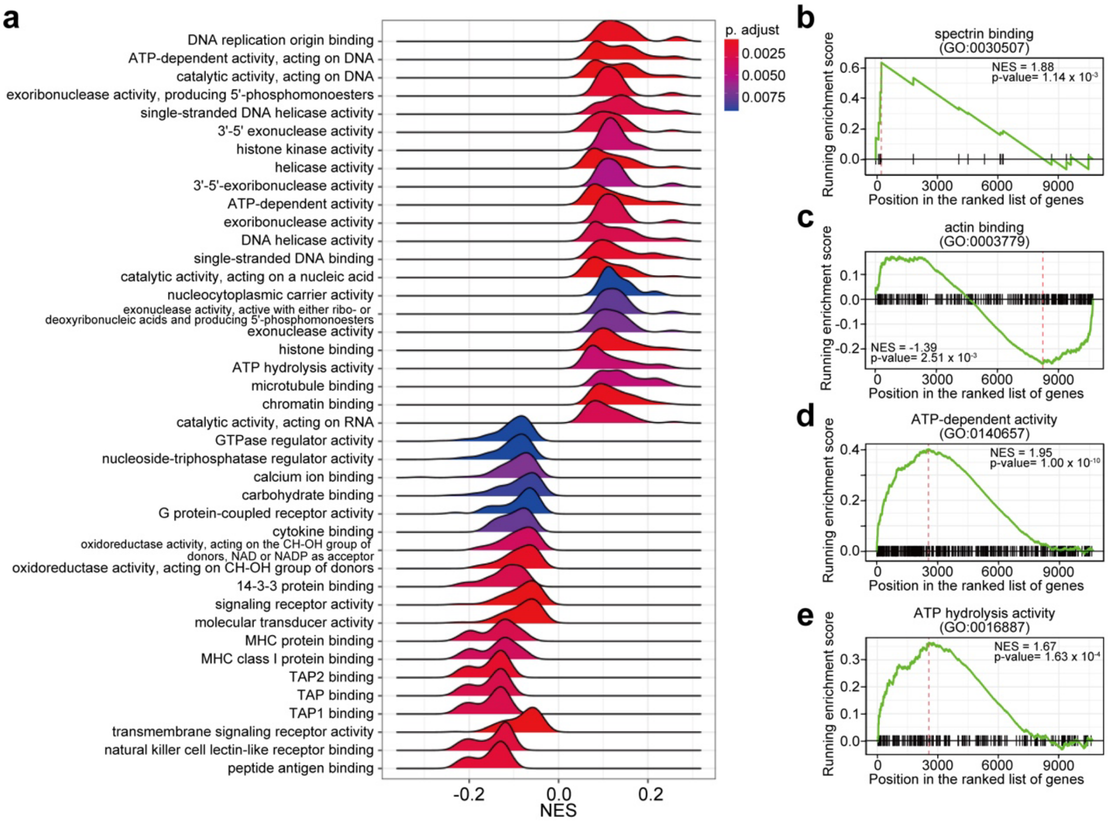

Supplementary Figure 10. **Gene set enrichment analysis on mHSPCs. a** Gene set enrichment analysis (GSEA) performed with the coefficient of correlation between the gene expression and the centred log ratio of 4 kDa DTD in the erythroid lineage (HSC-MPP-CMP-MEP/EryP-Erythroid-Erythrocyte). NES, normalized enrichment score. **b-e** GSEA showing enriched pathways with genes ordered by correlation shown in (*a*).

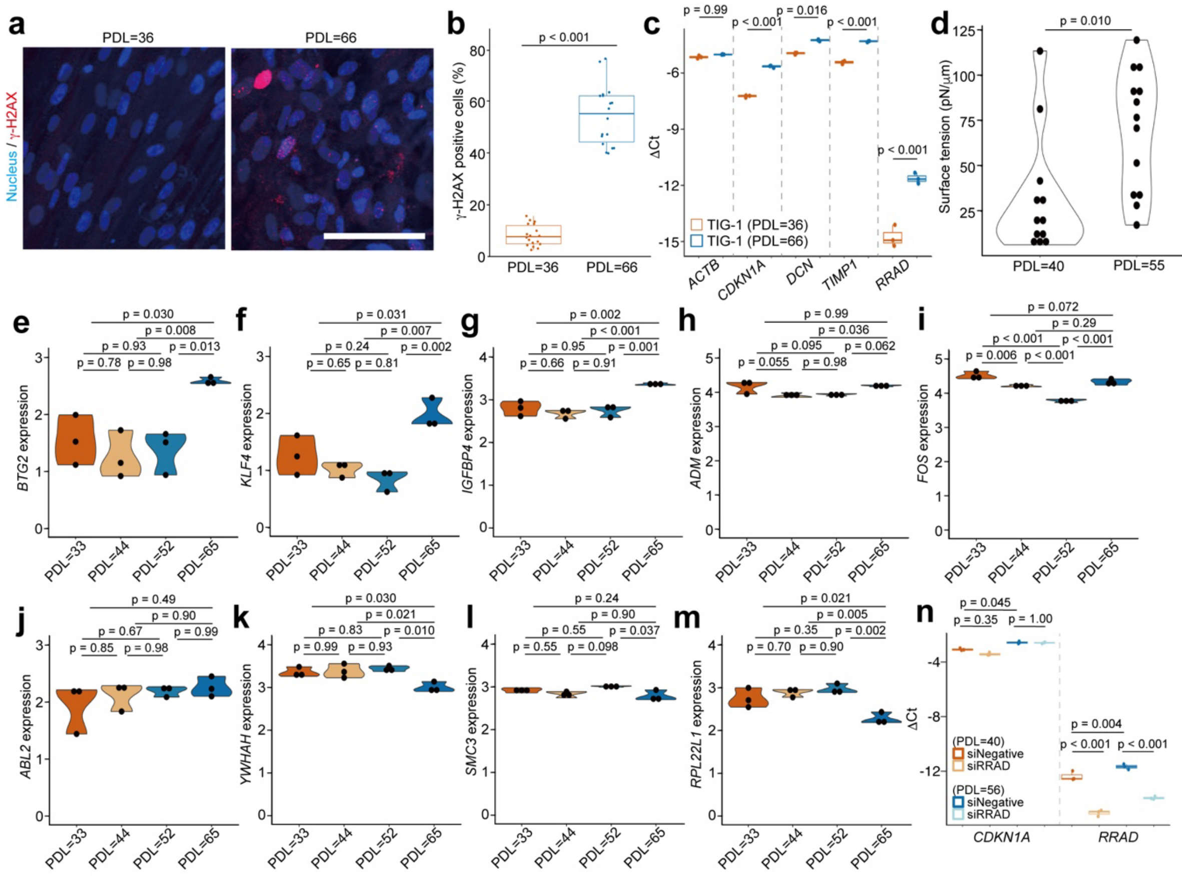

Supplementary Figure 11. **Senescent signature in TIG-1 cells. a** Fluorescence images of TIG-1 cells at different population doubling levels (PDL = 36 and 66) stained with anti-γH2AX antibody. Nuclei and antibodies were stained by Hoechst33342 (blue) and red-fluorescence secondary antibody (red), respectively. Scale bar, 100 µm. **b** Percentage of γH2AX-positive TIG-1 cells (PDL=36 and 66) (n = 18 independent images). Similar results were observed in independent duplicate experiments. **c** RT-qPCR analysis of five genes in TIG-1 cells (PDL=36 and 66). *GAPDH* mRNA was used to normalize the expressions (n = 3 biologically independent samples). **d** Median surface tension of young (PDL = 40; n =12 independent cells) and senescent (PDL=55; n =13 independent cells) TIG-1 cells measured by Atomic force microscopy (AFM) (independent cells examined in two independent experiments). **e-m** Comparison of gene expression in TIG-1 cells at different PDLs (PDL = 33, 44, 52, and 65) (n = 3 biologically independent samples). The genes were selected as shown in Fig. 3g (e: *BTG2*; f: *KLF4*; g: *IGFBP4*; h:*ADM*; i: *FOS*; j: *ABL2*; k: *YWHAH*; l: *SMC3*; and m:*RPL22L1*). **n** Expressions of *CDKN1A* and *RRAD* genes in TIG-1 cells (PDL=40 or 56) with or without the suppression of *RRAD* by siRNA. GAPDH mRNA was used to normalize the expressions (n = 3 biologically independent samples). For boxplot (b, c, and n), the centre line is the median, the box indicates the first and third quartiles, whiskers are minimum/maximum values excluding outliers, and dots are individual data. The P values (*p*) are indicated in the graph (b and d: two-tailed Student's t-test; c and e-n: Tukey's t-test). Source data are provided as a Source Data file.

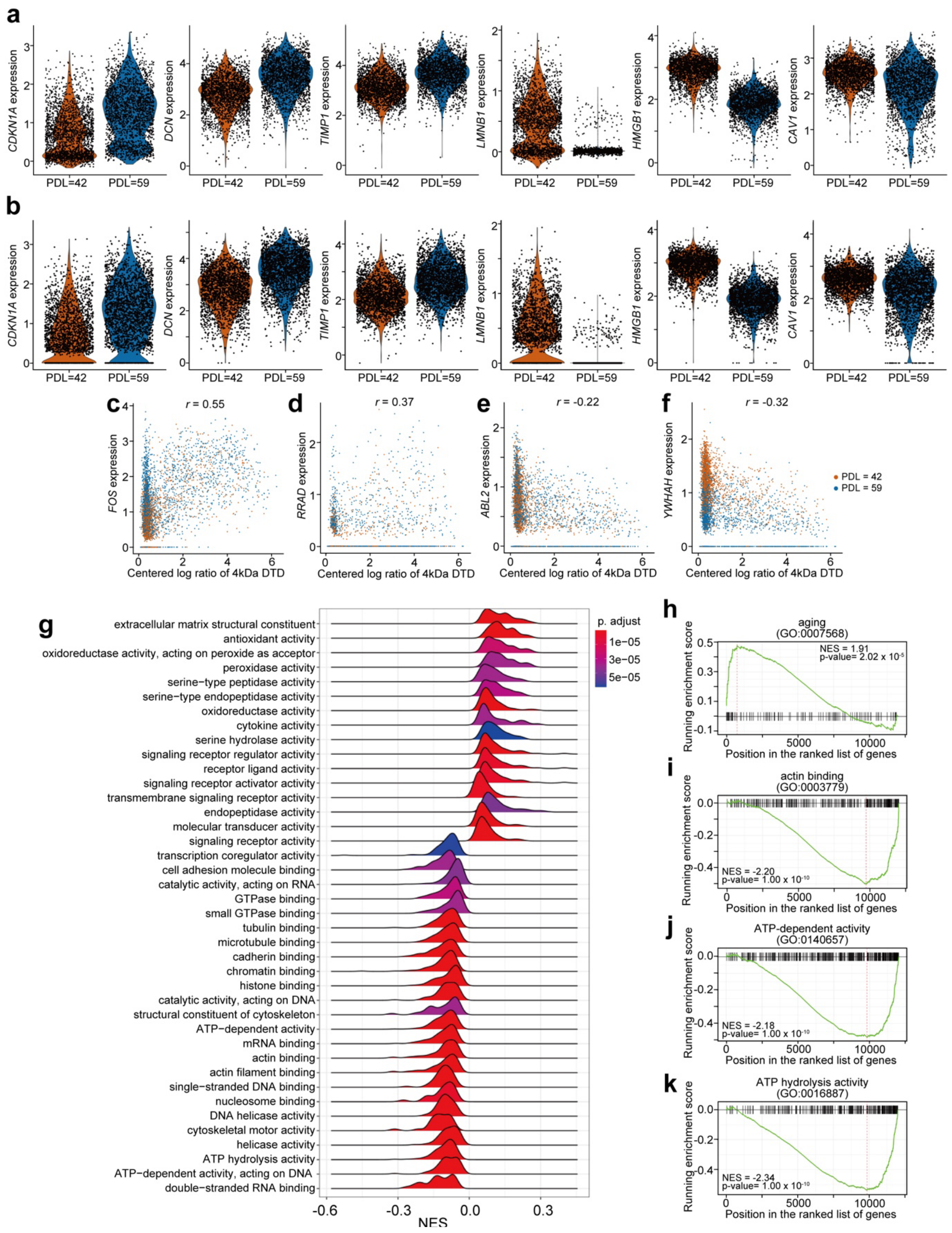

Supplementary Figure 12. **ELASTomics analysis of cellular senescence. a, b** Expression of senescence marker genes in control (*a*) and nanopore-electroporated (*b*) TIG-1 cells shown in Fig. 3c. **c-f** Scatterplot showing the relation between the centred log ratio of imported 4 kDa DTD (x-axis) and the expression levels of four genes (c: *FOS*; d: *RRAD*; e: *ABL2*; and f: *YWHAH*), which are positively or negatively correlated to the amount of imported 4 kDa DTD shown in Fig. 1g (y-axis) (orange: PDL = 42; blue: PDL = 59). **g** GSEA performed with the coefficient of correlation between the gene expression and centred log ratio of 4 kDa DTD in TIG-1 cells. NES, normalized enrichment score. **h-k** GSEA showing enriched pathways with genes ordered by correlation shown in (*g*). (*c-f*) Pearson's correlation coefficients (*r*) are indicated in the graph.

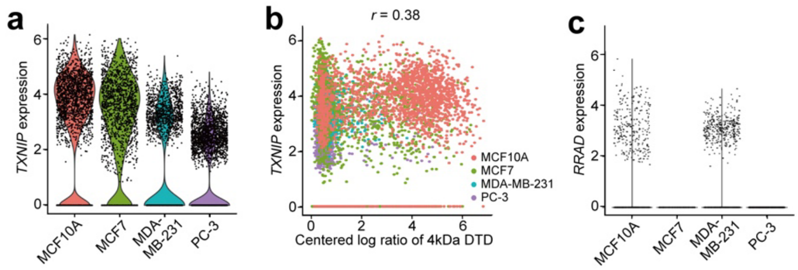

Supplementary Figure 13. **Glycolysis-related genes in cancer cell lines. a** Expression of *TXNIP* genes in nanopore-electroporated (40 V) four cancer cell lines. **b** Scatterplot showing the relation between the centred log ratio of imported 4 kDa DTD (x-axis) and the expression levels of *TXNIP*. The colour indicates the cell types (purple: PC-3; blue: MDA-MB-231; green: MCF7; red: MCF10A). **c** Expression of *RRAD* genes in nanopore-electroporated (40 V) four cancer cell lines. *RRAD* expression was undetectable in most of cancer cells (MCF10A: 12.8%; MCF7: 0.0%; MDA-MB-231: 15.3%; PC-3: 0.0%). (*b*) Pearson's correlation coefficient (*r*) is indicated in the graph.

Supplementary Table 1. Stokes radius and mobility of Dextran and DTD.

	Stokes radius (nm)	Electrophoretic mobility ($\times 10^{-9}$, m^2/Vs)	Primer concentration (μM)	Dextram concentration (mg/mL)
4 kDa_FITC-lysine-dextran	0.9±0.0	7.1±0.3	–	1.0
10 kDa_FITC-lysine-dextran	0.9±0.2	7.7±1.5	–	1.0
40 kDa_Antonia Red-lysine-dextran	2.0±0.2	0.0±0.0	–	1.0
70 kDa_FITC-lysine-dextran	8.9±3.5	1.4±0.6	–	1.0
150 kDa_Antonia Red-lysine-dextran	12.5±4.4	0.0±0.0	–	1.0
500 kDa_FITC-lysine-dextran	17.7±9.2	7.4±1.8	–	1.0
4 kDa_DTD	4.1±0.0	22.6±0.9	49.9	–
10 kDa_DTD	4.2±0.2	22.9±1.7	13.5	–
40 kDa_DTD	7.8±1.2	23.8±1.8	9.57	–
70 kDa_DTD	10.6±3.5	17.2±1.1	1.06	–
150 kDa_DTD	15.1±11.7	14.4±0.8	13.5	–
500 kDa_DTD	17.0±12.2	16.9±1.4	0.98	–

Supplementary Table 2. Primer information.

barcoded oligo-dT primers & TSO	sequence (Bio: Biotin-modulation)
ACCTCGTTGA	AAGCAGTGGTATCAACGCAGAGTACGACGCTCTTCCGATCTACCTCGTTGANNNNNNNNTTTTTTTTTTTTTTTTTTTTTTTTTTTTVN
CCATTGCGTT	AAGCAGTGGTATCAACGCAGAGTACGACGCTCTTCCGATCTCCATTGCGTTNNNNNNNNTTTTTTTTTTTTTTTTTTTTTTTTTTTTVN
ATCTCCGAAC	AAGCAGTGGTATCAACGCAGAGTACGACGCTCTTCCGATCTATCTCCGAACNNNNNNNNTTTTTTTTTTTTTTTTTTTTTTTTTTTTVN
GGCAGGTATT	AAGCAGTGGTATCAACGCAGAGTACGACGCTCTTCCGATCTGGCAGGTATTNNNNNNNNTTTTTTTTTTTTTTTTTTTTTTTTTTTTVN
AATCGTAGCG	AAGCAGTGGTATCAACGCAGAGTACGACGCTCTTCCGATCTAATCGTAGCGNNNNNNNNTTTTTTTTTTTTTTTTTTTTTTTTTTTTVN
GGTACTGCCT	AAGCAGTGGTATCAACGCAGAGTACGACGCTCTTCCGATCTGGTACTGCCTNNNNNNNNTTTTTTTTTTTTTTTTTTTTTTTTTTTTVN
GCCATTCTCC	AAGCAGTGGTATCAACGCAGAGTACGACGCTCTTCCGATCTGCCATTCTCCNNNNNNNNTTTTTTTTTTTTTTTTTTTTTTTTTTTTVN
TTACCGAGGC	AAGCAGTGGTATCAACGCAGAGTACGACGCTCTTCCGATCTTTACCGAGGCNNNNNNNNTTTTTTTTTTTTTTTTTTTTTTTTTTTTVN
TGGTCAGCCA	AAGCAGTGGTATCAACGCAGAGTACGACGCTCTTCCGATCTTGGTCAGCCANNNNNNNNTTTTTTTTTTTTTTTTTTTTTTTTTTTTVN
GATACGGAAC	AAGCAGTGGTATCAACGCAGAGTACGACGCTCTTCCGATCTGATACGGAACNNNNNNNNTTTTTTTTTTTTTTTTTTTTTTTTTTTTVN
TCGGCCTTAC	AAGCAGTGGTATCAACGCAGAGTACGACGCTCTTCCGATCTTCGGCCTTACNNNNNNNNTTTTTTTTTTTTTTTTTTTTTTTTTTTTVN
AGAACGTCTC	AAGCAGTGGTATCAACGCAGAGTACGACGCTCTTCCGATCTAGAACGTCTCNNNNNNNNTTTTTTTTTTTTTTTTTTTTTTTTTTTTVN
CCAGTTCCAA	AAGCAGTGGTATCAACGCAGAGTACGACGCTCTTCCGATCTCCAGTTCCAANNNNNNNNTTTTTTTTTTTTTTTTTTTTTTTTTTTTVN
GGCGTTAAGG	AAGCAGTGGTATCAACGCAGAGTACGACGCTCTTCCGATCTGGCGTTAAGGNNNNNNNNTTTTTTTTTTTTTTTTTTTTTTTTTTTTVN
CAACCGCTAA	AAGCAGTGGTATCAACGCAGAGTACGACGCTCTTCCGATCTCAACCGCTAANNNNNNNNTTTTTTTTTTTTTTTTTTTTTTTTTTTTVN
GAAGATCGAG	AAGCAGTGGTATCAACGCAGAGTACGACGCTCTTCCGATCTGAAGATCGAGNNNNNNNNTTTTTTTTTTTTTTTTTTTTTTTTTTTTVN
biotinylated template switching oligonucleotide (TSO)	[Bio]AAGCAGTGGTATCAACGCAGAGTACrGrG+G

SeqAmp PCR primers	sequence
primer2	AAGCAGTGGTATCAACGCAGAGT
additive primer	CCTTGGCACCCGAGAATTCCA

Indexing primers (TruSeq P5)	sequence
501	AATGATACGGCGACCACCGAGATCTACACTAGATCGCACACTCTTTCCCTACACGACGCTCTTCCGATCT
502	AATGATACGGCGACCACCGAGATCTACACCTCTCTATACACTCTTTCCCTACACGACGCTCTTCCGATCT
503	AATGATACGGCGACCACCGAGATCTACACTATCCTCTACACTCTTTCCCTACACGACGCTCTTCCGATCT
504	AATGATACGGCGACCACCGAGATCTACACAGAGTAGAACACTCTTTCCCTACACGACGCTCTTCCGATCT
505	AATGATACGGCGACCACCGAGATCTACACGTAAGGAGACACTCTTTCCCTACACGACGCTCTTCCGATCT
506	AATGATACGGCGACCACCGAGATCTACACACTGCATAACACTCTTTCCCTACACGACGCTCTTCCGATCT
507	AATGATACGGCGACCACCGAGATCTACACAAGGAGTAACACTCTTTCCCTACACGACGCTCTTCCGATCT
508	AATGATACGGCGACCACCGAGATCTACACCTAAGCCTACACTCTTTCCCTACACGACGCTCTTCCGATCT

Indexing primers (TruSeq Small RNA P7)	sequence
RPI1	CAAGCAGAAGACGGCATACGAGATCGTGATGTGACTGGAGTTCCTTGGCACCCGAGAATTCCA
RPI2	CAAGCAGAAGACGGCATACGAGATACATCGGTGACTGGAGTTCCTTGGCACCCGAGAATTCCA
RPI3	CAAGCAGAAGACGGCATACGAGATGCCTAAGTGACTGGAGTTCCTTGGCACCCGAGAATTCCA
RPI4	CAAGCAGAAGACGGCATACGAGATTGGTCAGTGACTGGAGTTCCTTGGCACCCGAGAATTCCA
RPI5	CAAGCAGAAGACGGCATACGAGATCACTGTGTGACTGGAGTTCCTTGGCACCCGAGAATTCCA
RPI6	CAAGCAGAAGACGGCATACGAGATATTGGCGTGACTGGAGTTCCTTGGCACCCGAGAATTCCA
RPI7	CAAGCAGAAGACGGCATACGAGATGATCTGGTGACTGGAGTTCCTTGGCACCCGAGAATTCCA
RPI8	CAAGCAGAAGACGGCATACGAGATTCAAGTGTGACTGGAGTTCCTTGGCACCCGAGAATTCCA

DTD oligonucleotide & Cell-hashing tag	Sequence (AmC6: amino-modulation)
4 kDa_DTD	[AmC6]CCTTGGCACCCGAGAATTCCAAACGTGATBAAAAAAAAAAAAAAAAAAAAAAAAAAAAAAAA
10 kDa_DTD	[AmC6]CCTTGGCACCCGAGAATTCCAAAACATCGBAAAAAAAAAAAAAAAAAAAAAAAAAAAAAAAA
40 kDa_DTD	[AmC6]CCTTGGCACCCGAGAATTCCAATGCCTAABAAAAAAAAAAAAAAAAAAAAAAAAAAAAAAAA
70 kDa_DTD	[AmC6]CCTTGGCACCCGAGAATTCCAAGTGGTCABAAAAAAAAAAAAAAAAAAAAAAAAAAAAAAAA
150 kDa_DTD	[AmC6]CCTTGGCACCCGAGAATTCCAACCACTGTBAAAAAAAAAAAAAAAAAAAAAAAAAAAAAAAA
500 kDa_DTD	[AmC6]CCTTGGCACCCGAGAATTCCAACATTGGCBAAAAAAAAAAAAAAAAAAAAAAAAAAAAAAAA
Cell-hashing tag_PC3	CCTTGGCACCCGAGAATTCCACATACCAABAAAAAAAAAAAAAAAAAAAAAAAAAAAAAAAA
Cell-hashing tag_MDA-MB-231	CCTTGGCACCCGAGAATTCCACAATGGAABAAAAAAAAAAAAAAAAAAAAAAAAAAAAAAAA
Cell-hashing tag_MCF7	CCTTGGCACCCGAGAATTCCACCGAAGTABAAAAAAAAAAAAAAAAAAAAAAAAAAAAAAAA
Cell-hashing tag_MCF10A	CCTTGGCACCCGAGAATTCCAGACTAGTABAAAAAAAAAAAAAAAAAAAAAAAAAAAAAAAA
Cell-hashing tag_75V	CCTTGGCACCCGAGAATTCCACACTTCGABAAAAAAAAAAAAAAAAAAAAAAAAAAAAAAAA
Cell-hashing tag_CytoD	CCTTGGCACCCGAGAATTCCACCAGTTCABAAAAAAAAAAAAAAAAAAAAAAAAAAAAAAAA

Supplementary Table 3. Concentration of DTD for each ELASTomics analysis.

	4 kDa DTD (μM)	10 kDa DTD (μM)	40 kDa DTD (μM)	70 kDa DTD (μM)	150 kDa DTD (μM)	500 kDa DTD (μM)	Cell-hashtag (μM)
TIG-1 cells	0.33	1.00	1.50	0.50	1.50	0.50	-
Mouse haematopoietic stem/progenitor cells	1.00	1.00	1.00	0.50	1.00	0.50	-
PC-3, MDA-MB-231, MCF7, MCF10A cells	1.00	1.00	1.00	0.50	1.00	0.50	1.00

Supplementary Methods

Theoretical analysis on the DTD translocation via electroporation and electrophoresis

In the following section, to correlate the amount of the imported macromolecules into cells via nanopore-electroporation and electrophoresis to the surface tension of the lipid bilayer, we first discuss the relation between the surface tension and the radius of a pore formed in a lipid bilayer. We second estimate the amount of the macromolecules translocated through the pore via electrophoresis considering the hindrance in the transport of macromolecules due to the non-negligible Stokes radius of the macromolecules (3 nm-20 nm) relative to the radius of the pore (smaller than 20 nm) in a lipid bilayer.

First, the change in the free energy of the plasma membrane by the formation of a hydrophilic pore of radius *r* at a transmembrane voltage of magnitude *U* is written as[1]

$$\Delta W = 2\pi r\gamma\left(1+\frac{C}{r^5}\right) - \pi r^2\sigma - \frac{(\varepsilon_e-\varepsilon_m)\pi r^2}{2d}U^2, \quad (S1)$$

where C, d, ε_e, ε_m, γ and σ are respectively 1.39×10^{-46} J/m^4, the thickness of the plasma membrane, the permittivity of the extracellular medium, the permittivity of the plasma membrane, the line tension of the peripheral of a pore, and the surface tension (Supplementary Fig. 5a). The evolution of pores in a lipid bilayer is modelled by the Smoluchowski equation as

$$\frac{\partial n}{\partial t} = D_p\frac{\partial}{\partial r}\left(\frac{\partial n}{\partial r} + \frac{n}{kT}\frac{\partial \Delta W(r,\gamma,\sigma,U)}{\partial r}\right), \quad (S2)$$

where D_p, k and T are the pore diffusion coefficient, the Boltzmann constant, and the temperature. In the time scale of the electrophoresis (~ms), the Smoluchowski equation reaches the steady state, and the distribution of the radius of pores in a lipid bilayer $n(r, \gamma, \sigma, U)$ is given by the Boltzmann distribution as

$$n(r,\gamma,\sigma,U) = n_0\exp\left(-\frac{\Delta W(r,\gamma,\sigma,U)}{kT}\right), \quad (S3)$$

where n_0 is a scaling factor (Supplementary Fig. 5b).

Second, the flux of the macromolecules through the pore via electrophoresis is calculated by:

$$j_{DTD} = -H_{DTD}\mu_{DTD}\frac{U}{d}c_{DTD}, \quad (S4)$$

where c_{DTD}, H_p and μ_{DTD} are respectively the concentration of the macromolecule, the hindrance factor, and the electrophoretic mobility. The hindrance factor is given as[2]:

$$H_{DTD} = (1-\lambda)^2\frac{6\pi}{f_t(\lambda)}, \quad (S5)$$

$$f_t(\lambda) = \frac{9}{4}\pi^2\sqrt{2}(1-\lambda)^{-\frac{5}{2}}\left(1-\frac{73}{60}(1-\lambda)+\frac{77293}{50400}(1-\lambda)^2\right) -22.5083 - 5.6117\lambda - 0.3363\lambda^2 - 1.216\lambda^3 + 1.647\lambda^4, \quad (S6)$$

where λ is r_{DTD}/r_p. r_{DTD} and r_p are respectively the Stokes radius of the macromolecules and the radius of the pore in a lipid bilayer. Thus, the expected amount of the macromolecules transported through the pore for $\Delta\tau$ can be estimated by

$$q_{DTD}(\gamma,\sigma,U) = \int_{r_{DTD}}^{r_{max}} j_{DTD}(r_p)\pi r_p^2 n(r_p,\gamma,\sigma,U)dr_p\Delta\tau, \quad (S7)$$

where r_{max} is the critical radius of a reversible pore, $r_{max}=\gamma/\sigma$. The final equation predicts that the amount of the macromolecules transported via nanopore-electroporation and

electrophoresis is a function of the line tension, the surface tension, and the transmembrane voltage of the lipid bilayer. The amount also depends on r_{DTD}, especially through j_{DTD}.
To characterize q_{DTD}, we computed the relative permeability, $q_{\mathrm{DTD}}/(n_0 \mu_{\mathrm{DTD}} c_{\mathrm{DTD}} U \Delta \tau / d)$, at various σ and r_{DTD} while assuming that γ is a constant using the parameters summarized in Supplementary Table 4 (Supplementary Fig.5c, d).

Supplementary Table 4. Parameters for the theoretical analysis of nanopore-electroporation and electrophoresis[1]

Parameters	Values
Permittivity of extracellular medium ε_e	7.1×10^{-10} F/m
Permittivity of lipid bilayer ε_m	4.4×10^{-11} F/m
Thickness of lipid bilayer d	5×10^{-9} m
Line tension of peripheral of pore in a lipid bilayer γ	2×10^{-11} J/m
Temperature	300 K
Voltage U	50 mV

Comparison of FITC-BSA and the DTD counting with index sorting of single cells

We purchased barcoded oligo-dT primers containing 8 nt long UMI and 10 nt long cellular barcodes, which have 50-60 GC% (Supplementary Table 2). Nanopore-electroporated single cells were sorted into 96-well plates containing 4.25 µL of lysis buffer (1.12 x Lysis buffer (Takara), 0.23 unit/µL of RNase inhibitor (Takara)) and 1.2 µM of barcoded oligo-dT primers using a FACSAria™ II Cell Sorter (BD Biosciences). During single-cell sorting, the 96-well plate was kept at 4˚C. Immediately after the single-cell sorting, the plate was sealed with MicroAmp™ Optical Adhesive Film (Applied Biosystems™), centrifuged at 10,000 g and 4˚C for 1 min, snap-frozen on dry ice and stored at −80°C until processing.
On the day of processing, the plate was thawed on ice and incubated at 25˚C for 5 min. To denature RNA, the plate was heated at 72 ˚C for 3 min and immediately placed on ice for 2 min. We peeled off the sealing film and added 5.75 µL of RT mix (1xFirst-Strand Buffer, 2.4 µM biotinylated template switching oligonucleotide (TSO, Qiagen), 1 mM dNTP mix, 2 mM DTT, 1.1 unit/µL RNase inhibitor, 10 units/µL SMARTScribe Reverse Transcriptase (Takara) to each well. We sealed the plates again, agitated them at 900 rpm and 4˚C for 20 s, and then briefly centrifuged them. The plates were incubated in a thermal cycler at 42˚C for 90 min to obtain first-strand cDNA by reverse transcription and then heated at 70˚C for 10 min to stop the reaction. To remove excess RT primers, we added 1 µL of 2.5 U Exonuclease I (Takara) and incubated at 37˚C for 30 min, followed by inactivation of heating at 80˚C for 20 min. The first-strand cDNA was amplified in a 25 µL reaction containing 0.24 µM primer2, 9 nM additive primer (Supplementary Table 2), 1xSeqAmp PCR Buffer and 0.025 units/µL SeqAmp DNA Polymerase (Takara). The plate was heated for the PCR at 95˚C for 1 min; 15 cycles of 98˚C for 10 s and 65˚C for 30 s; 68˚C for 4 min;72˚C for 10 min. The PCR products from 16 wells were pooled together. We separated the mRNA-derived cDNAs (long cDNA) and the DTD-derived cDNAs (<200bp short cDNA) using SPRIselect beads. To bind long cDNA on SPRI beads, we added 240 µL of SPRI beads (0.6x) to 400 µL of the pooled sample of 16 single cells in a 1.5 mL tube. We transferred the supernatant containing the DTD to a new tube and added 560 µL of SPRI beads. The magnetic beads were washed twice on a magnetic stand with 1 ml of fresh 80% (vol/vol) ethanol and air-dried for 2.5 min at room temperature. The cDNA derived from DTD was eluted with 11 µL of elution buffer (10 mM Tris-HCl, pH 8.5).

We discarded the DNA products derived from mRNA. We constructed the DTD library using the same protocol described in the library construction of ELASTomics. We assessed the yield and length of DTD library respectively with a Qubit™ dsDNA HS Assay Kits and the Agilent High Sensitivity DNA Kit (Agilent). We also quantified the library with the KAPA Library Quantification Kits (Roche) according to the manufacturer's protocol. The library was sequenced using HiSeq X (Illumina) instrument with 2 x 150 bp paired-end reads.

Numerical simulation on a focused electric field in the vicinity of the nanopores

To visualize the focused electric field in the vicinity of the nanopore, we simulated the electric field in our experimental system using COMSOL Multiphysics finite-element-analysis software (COMSOL Inc) along with the AC/DC Module (steady state). We assumed each nanopore is an independent system and identical to the others, and thus we modelled the single nanopore as an axisymmetric cylinder with 25-μm height and 100 nm in diameter filled with the HEPES based buffer. The PBS in a culture chamber (on top of the track-etched membrane, 0.633 Ωm) and the HEPES based buffer in the electrode holder (under the track-etched membrane, 44.8 Ωm) were both simplified as cylindrical media with 1-mm height and 0.41-μm diameter, which corresponded to the average distance between nanopores. We analytically calculated the voltage difference between the upper and lower boundaries of the simulated area. Assuming that the top and bottom electrodes (in the arrangement shown in Supplementary Fig. 2f) were subjected to electric potentials of 40 V and 0 V, respectively, the electric potentials at the upper and lower boundary of the simulated area were estimated as 38.6 V and 27.5 V, respectively. We thus calculated an applied voltage of 11.1 V between the top and the bottom boundaries. Boundary surfaces other than the top and bottom were assumed to be insulators. We build the mesh through a physics-controlled mesh sequence type with the parameters of 'Extremely fine' element size, 0.1-μm of maximum element size, and 0.0205-μm of minimum element size. The numerical result showed the focused electric field rapidly decays as the distance from the nanopore within the order of 1 μm.

1. Kotnik, T., Rems, L., Tarek, M. & Miklavcic, D. Membrane Electroporation and Electropermeabilization: Mechanisms and Models. *Annual Review of Biophysics* **48**, 63-91 (2019).
2. Mukherjee, P., Nathamgari, S.S.P., Kessler, J.A. & Espinosa, H.D. Combined Numerical and Experimental Investigation of Localized Electroporation-Based Cell Transfection and Sampling. *ACS Nano* **12**, 12118-12128 (2018).
