# Supplementary material for: High-throughput mechanical phenotyping and transcriptomics of single cells
Source: Nat Commun. 2024 May 17;15:3812. doi: 10.1038/s41467-024-48088-5 (PMC11101642; doi:10.1038/s41467-024-48088-5)
Supplement: Supplementary file 3 — Reporting Summary [file 41467_2024_48088_MOESM3_ESM.pdf]

nature portfolio

Corresponding author(s): Hirofumi Shintaku

Last updated by author(s): 2024/04/11

Reporting Summary

Nature Portfolio wishes to improve the reproducibility of the work that we publish. This form provides structure for consistency and transparency in reporting. For further information on Nature Portfolio policies, see our Editorial Policies and the Editorial Policy Checklist.

Statistics

For all statistical analyses, confirm that the following items are present in the figure legend, table legend, main text, or Methods section.

n/a	Confirmed	
[ ]	[x]	The exact sample size (n) for each experimental group/condition, given as a discrete number and unit of measurement
[x]	[ ]	A statement on whether measurements were taken from distinct samples or whether the same sample was measured repeatedly
[ ]	[x]	The statistical test(s) used AND whether they are one- or two-sided<br>*Only common tests should be described solely by name; describe more complex techniques in the Methods section.*
[x]	[ ]	A description of all covariates tested
[ ]	[x]	A description of any assumptions or corrections, such as tests of normality and adjustment for multiple comparisons
[ ]	[x]	A full description of the statistical parameters including central tendency (e.g. means) or other basic estimates (e.g. regression coefficient) AND variation (e.g. standard deviation) or associated estimates of uncertainty (e.g. confidence intervals)
[ ]	[x]	For null hypothesis testing, the test statistic (e.g. F, t, r) with confidence intervals, effect sizes, degrees of freedom and P value noted<br>*Give P values as exact values whenever suitable.*
[x]	[ ]	For Bayesian analysis, information on the choice of priors and Markov chain Monte Carlo settings
[x]	[ ]	For hierarchical and complex designs, identification of the appropriate level for tests and full reporting of outcomes
[ ]	[x]	Estimates of effect sizes (e.g. Cohen's d, Pearson's r), indicating how they were calculated

Our web collection on statistics for biologists contains articles on many of the points above.

Software and code

Policy information about availability of computer code

Data collection	Microscope data were acquired by FV10-ASW4.2(Olympus FV1000).Flow cytometry data were acquired by Attune Cytometric Software v1.2 (Applied Biosystem). qPCR data were acquired by Stephen Software v2.3 (Applied Biosystem). FE-SEM data were acquired by Hitachi SU8200 series Scanning Electron Microscope program ver. 3.18 (Hitachi SU8200). AFM data were acquired by Igor Pro Ver.6.0.4.0 and MFP-3D_080501+1804C (WaveMetrics). 10x Genomics (cellranger version 6.1.2), Seurat (R, version 4.0.4 package), and custom codes are used for the single-cell RNA sequencing, AFM analysis, and flow cytometry analysis. Source code and analysis scripts are updated in the branch of “Shiomi.et.al2023” at https://github.com/RIKEN-Microfluidics-Lab/ELASTomics.git.
Data analysis	ImageJ (Java 1.8.0) was used for imaging analysis. FlowJo (version 10.8.1) and R (version 4.2.2) was used for the statistical analysis. Atomic force microscopy data were analyzed by analysis scripts are updated in the branch of “Shiomi.et.al2023” at https://github.com/RIKEN-Microfluidics-Lab/ELASTomics.git.

For manuscripts utilizing custom algorithms or software that are central to the research but not yet described in published literature, software must be made available to editors and reviewers. We strongly encourage code deposition in a community repository (e.g. GitHub). See the Nature Portfolio guidelines for submitting code & software for further information.

Data

Policy information about availability of data

All manuscripts must include a data availability statement. This statement should provide the following information, where applicable:

- Accession codes, unique identifiers, or web links for publicly available datasets
- A description of any restrictions on data availability
- For clinical datasets or third party data, please ensure that the statement adheres to our policy

Raw and assembled sequencing data from this study have been deposited in NCBI's Sequence Read Archive (SRA) under accession code PRJNA928498 [https://www.ncbi.nlm.nih.gov/bioproject/928498] and PRJNA928499 [https://www.ncbi.nlm.nih.gov/bioproject/928499]. Electron microscopy images from this study have been deposited in Systems Science Biological Dynamics repository (SSBD:repository) under accession code ssbd-repos-000343 [https://doi.org/10.24631/ssbd.repos.2024.04.343]. Flow cytometry data from this study have been deposited in GitHub under accession code 10.5281/zenodo.10934353 [https://zenodo.org/doi/10.5281/zenodo.10934352]. Other source data are provided Supplementary Information/Source Data file.

Research involving human participants, their data, or biological material

Policy information about studies with human participants or human data. See also policy information about sex, gender (identity/presentation), and sexual orientation and race, ethnicity and racism.

Reporting on sex and gender	N/A
Reporting on race, ethnicity, or other socially relevant groupings	N/A
Population characteristics	N/A
Recruitment	N/A
Ethics oversight	N/A

Note that full information on the approval of the study protocol must also be provided in the manuscript.

Field-specific reporting

Please select the one below that is the best fit for your research. If you are not sure, read the appropriate sections before making your selection.

☒ Life sciences ☐ Behavioural & social sciences ☐ Ecological, evolutionary & environmental sciences

For a reference copy of the document with all sections, see nature.com/documents/nr-reporting-summary-flat.pdf

Life sciences study design

All studies must disclose on these points even when the disclosure is negative.

Sample size	This sample size was chosen based on preliminary experiments indicating that it would be sufficient to detect significant differences in mean.
Data exclusions	No exclusion criteria were pre-established and all data were included.
Replication	Number of replicates stated in the figure legends where applicable.
Randomization	Wild-type mice were randomly selected for the experiments. The cell lines were randomly seeded for in vitro assays.
Blinding	N/A. Blinding is not relevant since sample information can be identified by the hash-tag primers.

Reporting for specific materials, systems and methods

We require information from authors about some types of materials, experimental systems and methods used in many studies. Here, indicate whether each material, system or method listed is relevant to your study. If you are not sure if a list item applies to your research, read the appropriate section before selecting a response.

Materials & experimental systems

n/a	Involved in the study
☐	☒ Antibodies
☐	☒ Eukaryotic cell lines
☒	☐ Palaeontology and archaeology
☐	☒ Animals and other organisms
☒	☐ Clinical data
☒	☐ Dual use research of concern
☒	☐ Plants

Methods

n/a	Involved in the study
☒	☐ ChIP-seq
☐	☒ Flow cytometry
☒	☐ MRI-based neuroimaging

Antibodies

Antibodies used

We used 10 ng/µL TotalSeq™-A0130 anti-mouse Ly-6A/E (Sca-1) Antibody (10814, Biolegend), TotalSeq™-A0012 anti-mouse CD117 (c-kit) Antibody (105843, Biolegend), TotalSeq™-A0203 anti-mouse CD150 (SLAM) Antibody (115945, Biolegend), and TotalSeq™-A0429 anti-mouse CD48 Antibody (103447, Biolegend) for CITE-seq experimence. c-kit (CD117) micro beads (130-097-146, 1:5, Miltenyi Biotec) were used to isolate mouse bone marrow (BM) cells from mice femurs. 10 ng/µL Pacific Blue™ anti-mouse TER-119 Antibody (116232, Biolegend) were used for staining the erythroid progenitor cells. Anti-gammaH2AX mouse antibody (G266, 1:50, Dojindo) and secondary goat antibody (G266, 1:50, Dojindo) were used for the DNA damage detection along cellular senescence.

Validation

According to the manufacturer's website, the antibodies are quantify control tested. TotalSeq antibodies that showed patterned staining were considered as valid.
The manufacture's validation data are available on their websites:G266, https://www.dojindo.com/manual/G265_G266_G267/

Eukaryotic cell lines

Policy information about cell lines and Sex and Gender in Research

Cell line source(s)

MCF10A (CRL-10317), MCF7 (HTB-22), and MDA-MB-231 (HTB-26) were newly purchased from ATCC. TIG-1 (JCRB0501 and JCRB0504) were also purchased from JCRB. PC-3 (RCB2145), K562 (RCB0027), CHO-K1 (RCB0285), HeLa (RCB0007), OVCAR-3 (RCB2135), and GEM-81 (RCB1174) were purchased from RIKEN BRC.

Authentication

Cells were not authenticated.

Mycoplasma contamination

We regularly tested for mycoplasma contamination using a CycleavePCRTM Mycoplasma Detection Kit (TAKARA) and no positive sign of mycoplasma contamination for these cell lines.

Commonly misidentified lines
(See ICLAC register)

No commonly misidentified lines were used.

Animals and other research organisms

Policy information about studies involving animals; ARRIVE guidelines recommended for reporting animal research, and Sex and Gender in Research

Laboratory animals

8-week old male C57BL/6J mice (JSLC Co., Hamamatsu, Japan) were purchased. The animal facilities were maintained at 20–25°C with 40–60% humidity under a standard 12-h light–dark cycle.

Wild animals

This study did not used wild animals.

Reporting on sex

Male mice were used in the experiments.

Field-collected samples

This study did not involve samples collected from the fields.

Ethics oversight

All of the experiments were approved by the accordance with the guidelines for animal experimentation of the RIKEN.

Note that full information on the approval of the study protocol must also be provided in the manuscript.

Plants

Seed stocks	N/A
Novel plant genotypes	N/A
Authentication	N/A

Flow Cytometry

Plots

Confirm that:

- [x] The axis labels state the marker and fluorochrome used (e.g. CD4-FITC).
- [x] The axis scales are clearly visible. Include numbers along axes only for bottom left plot of group (a 'group' is an analysis of identical markers).
- [ ] All plots are contour plots with outliers or pseudocolor plots.
- [ ] A numerical value for number of cells or percentage (with statistics) is provided.

Methodology

Sample preparation	After nanopore-electroporation, the adherent cells were detached from the track-etched membrane with trypsin (Nacalai tesque). Cells were washed with PBS via centrifugation at 390 g for 3 min. Mouse HSPCs were incubated in PBS (0.1% BSA) including 10 mg/L Pacific Blue" anti-mouse TER-119 Antibody (116232, Biolegend) on ice for 30 min and washed with PBS (0.1% BSA). To detect live/dead cells, cells were incubated for 10 min in PBS containing 5 g/mL propidium iodide and 5 g/mL Hoechst 33342 at room temperature.
Instrument	Attune acoustic focusing cytometer (Applied Biosystem)
Software	FlowJo v10(BD)
Cell population abundance	Pure cell lines were used at desired proportions for cell line experiments. Mouse bone marrow (BM) cells isolated from mice femurs were bound to c-kit (CD117) micro beads (Miltenyi Biotec) and then collected by LS column (Miltenyi Biotec).
Gating strategy	Debris and aggregated cells were removed with the initial FSC/SSC gate. Hoechst33342-negative or PI-positive cells were also excluded from the analysis.

- [ ] Tick this box to confirm that a figure exemplifying the gating strategy is provided in the Supplementary Information.
